# Benzodiazepines for Treatment-Resistant Major Depressive Disorder and Obsessive-Compulsive Disorder With Comorbid Mega Cisterna Magna

**DOI:** 10.7759/cureus.46670

**Published:** 2023-10-08

**Authors:** Arifah Ismail, Asrenee Ab Razak, Khairil Amir Sayuti, Picholas Kian Ann Phoa

**Affiliations:** 1 Department of Psychiatry, Hospital Universiti Sains Malaysia, Kota Bharu, MYS; 2 Department of Psychiatry, Universiti Sains Malaysia School of Medical Sciences, Kota Bharu, MYS; 3 Department of Radiology, Hospital Universiti Sains Malaysia, Kota Bharu, MYS; 4 Department of Radiology, Universiti Sains Malaysia School of Medical Sciences, Kota Bharu, MYS

**Keywords:** benzodiazepine use, cerebellum, mega cisterna magna, obsessive-compulsive disorder, major depressive disorder

## Abstract

This article discusses the case of an adult woman with comorbid major depressive disorder (MDD) and obsessive-compulsive disorder (OCD). Due to her poor response to initial treatment regimens, a brain computed tomography (CT) was performed, revealing mega cisterna magna (MCM). Subsequently, she responded well to the addition of lorazepam, a benzodiazepine, along with fluvoxamine and quetiapine XR. The clinical significance of MCM and MDD-OCD remains partially understood. Thus, this case report aims to contribute to the literature and review the treatment of benzodiazepines in relation to MCM, MDD, and OCD comorbidities.

## Introduction

Accumulating evidence and recent findings have presented the cerebellum’s broader functions. The cerebellum appears to play a significant role in regulating cognitive and emotional processes, as well as in the control of balance and intentional voluntary movements [1]. For instance, Schmahmann and colleagues characterized the “cerebellar cognitive affective syndrome (CCAS)” or “Schmahmann syndrome” as a constellation of cerebellar-induced sequalae comprising deficits in the cognitive domains of executive function, spatial cognition, and language, as well as neuropsychiatric presentations like impulsivity, stereotypical behaviors, illogical thoughts, obsessive behaviors, dysphoria, and depression [2]. After decades of investigation, the cerebellum plays a crucial role in the pathophysiology of psychiatric disorders [3].

Mega cisterna magna (MCM) is a cystic posterior fossa malformation that is formed embryologically from the permeabilization of Blake’s pouch and is characterized by an enlarged cisterna magna with intact cerebellar vermis and the absence of hydrocephalus [4]. While the majority of MCM cases were incidental radiological findings, studies suggest a potential association with psychiatric disorders and cognitive impairment [4]. Most published cases reported patients with psychotic spectrum disorders, including schizophrenia, catatonic schizophrenia, shared psychotic disorder, and bipolar mood disorder [5-9]. Only one case reported an incidental finding of MCM in a schizophrenic patient with co-morbid obsessive-compulsive disorder (OCD) [10, 11]. In this case, Kani and colleagues proposed that clonazepam, a benzodiazepine, may be an effective treatment for treatment-resistant obsessive-compulsive presentation. They hypothesized that clonazepam may improve GABAergic function and inhibitory processes by GABAergic neurons situated mostly in the cerebellum and neocortex [11]. However, according to our current knowledge, there has yet to be any case of OCD-MDD comorbidity with MCM reported elsewhere. Thus, this paper aims to contribute to the literature and present a case study involving a patient with comorbid OCD-MDD with MCM who was effectively treated with lorazepam.

## Case presentation

A female teacher in her late 50s visited our psychiatry outpatient clinic complaining of sleeping difficulties for the past two months. Her problems started eight months before her first appointment with a psychiatrist while she was getting ready for her pilgrimage (Umrah) in Mecca. During this time, she developed an obsessive concentration on cleanliness and showed increased particularity about her surroundings' cleanliness, which caused frequent intrusive thoughts about contamination. She would firmly refuse to sit in seats, as her family members had noticed. However, the patient’s intrusive thoughts became more frequent when performing Umrah, which drove her to continually wash her clothes and sandals and engage in other obsessive cleaning activities. She spent a lot of time cleaning, making it difficult to complete her religious duties during Umrah. Additionally, she experienced recurrent, intrusive thoughts that her spiritual efforts were insufficient to atone for her previous sins and that God had not pardoned her for them. She continued to perform religious rites to find solace. She neglected her meals and adhered to other daily tasks because she was preoccupied with religious issues.

Her condition deteriorated when she returned from Umrah, interfering with her regular cleaning and teaching responsibilities. She started having trouble falling asleep, and occasionally, these upsetting thoughts would cause her to wake up at midnight. She attempted to deal with these thoughts by reciting prayers. She recognized the intrusive ideas as her own and did not perceive them as coming from an external source. A month before her psychiatric visit, she started feeling down almost daily and no longer enjoyed doing her daily activities. She found it challenging to focus on her tasks as a teacher. Additionally, she has become more reclusive, spending more time alone in her room and interacting with her family less. In contrast to her sleep patterns of six to seven hours each night, she now sleeps only about four hours. She reported feeling fatigued and having less energy in the mornings. Her appetite was also affected, as she now eats only one meal daily.

Otherwise, she denied having symptoms of hallucination and delusion. She had never experienced excessive, uncontrollable anxiety or worry about daily activities or events. There is no history of sudden unresponsiveness, blank stares, seizures, or abnormal body movement. She has no record of substance abuse. She was previously healthy with no known comorbidities. Her medical and psychiatric history during infancy and childhood was also insignificant, with normal psychomotor development. She also denied any family history of neuropsychiatric disorders.

Upon mental state examination, she was a moderately built Malay lady dressed decently and cleanly. She appeared to be restless and was not cooperative throughout the interview. Rapport and eye contact were not established. She spoke in Malay coherently and relevantly but with a reduced amount, rate, and tone. She described her mood as anxious, whereas her attitude was anxious and restricted. She was obsessed with her perceived sinfulness and preoccupied with her thoughts on returning home. Otherwise, she denied delusions, hallucinations, and suicidal thoughts. She had poor attention and concentration. However, the patient refused further cognitive assessment after several attempts.

**Figure 1:**
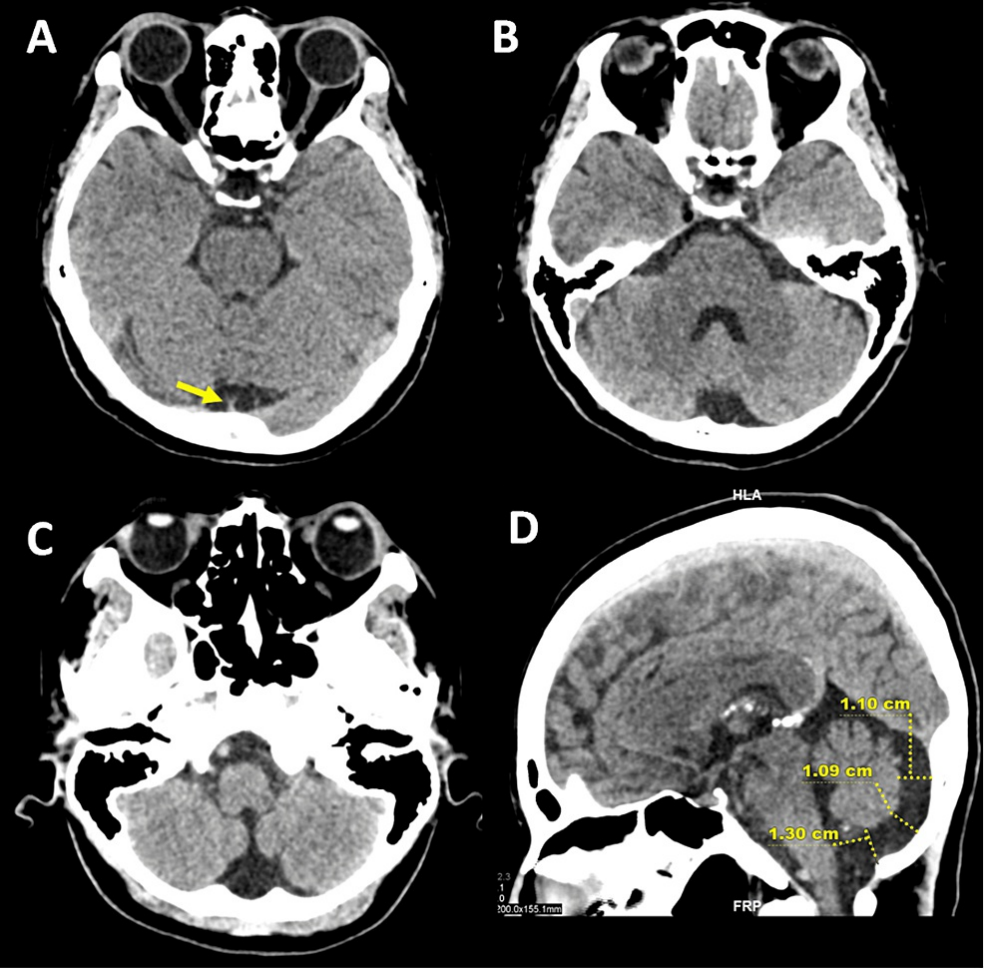
(A, B, C) A non-contrast CT brain in the axial plane reveals an enlarged retrocerebellar CSF space with normal vermis, cerebellar hemispheres, and a fourth ventricle. There was no hydrocephalus or mass effect on the cerebellum. Falx cerebelli is undisplaced (arrow). (D) The midsagittal plane demonstrates the diameter of the cisterna magna; the maximum measurement was obtained from the posterior lip of the foramen magnum to the caudal margin of the inferior vermis. There is no upward sloping of the tentorium cerebelli.

She was then diagnosed with OCD and MDD per the Diagnostic and Statistical Manual of Mental Disorders, Fifth Edition (DSM-5). Treatment was initiated with quetiapine, up to 600mg, and mirtazapine, up to 45mg. However, there was no significant improvement with increasing dosages. Thus, a computed tomography (CT) scan of the brain was done to rule out organicity, thus revealing normal brain parenchyma. However, the retrocerebellar cerebrospinal fluid space (CSF) was dilated with a maximum diameter of 1.30cm, in keeping with MCM. The vermis, cerebellar hemispheres, and fourth ventricle were normal in size. There was no evidence of hydrocephalus, displacement of the tentorium and falx cerebelli, or corpus callosum agenesis. The posterior fossa was also not enlarged (Figure 1).

Nonetheless, the patient experienced some relief from symptoms with the occasional use of alprazolam 0.25mg. Mirtazapine was gradually tapered down and replaced with fluvoxamine, which was increased to 150mg O.N. Lorazepam 1mg B.D. was added to the treatment regimen, and the patient responded positively. She experienced overall improvements in sleep, appetite, social interaction, and the ability to perform household chores. However, after approximately two years and five months on this medication regimen, the patient disclosed her concern about the perceived excessive medication consumption, therefore self-adjusting her medication regimen. She claimed her symptoms were well-maintained. Upon her last follow-up assessment, the regimen was adjusted to fluvoxamine 100mg O.N., quetiapine XR 600mg O.N., and lorazepam 1mg O.N. to address her worries about medication burden while ensuring effective treatment. She was advised to comply with the medication regimen and subsequent follow-up sessions.

## Discussion

Our case study presented manifestations of depressive symptoms and obsessive-compulsive behaviors associated with MCM. MCM is diagnosed when the maximum diameter of the retrocerebellar CSF space exceeds 1.0cm in the midsagittal plane. The normal size of the cerebellum, vermis, fourth ventricle, and corpus callosum, as well as undisplaced tentorium cerebelli in this patient, exclude other common differentials such as cerebellar hypoplasia/atrophy and Dandy-Walker malformation, although the latter is usually diagnosed in children of pediatric age. A common mimicker is an arachnoid cyst that typically exerts a mass effect on the cerebellum with a deviation of falx cerebelli, features that are absent in our CT. Blake pouch cyst is a rarely diagnosed infracerebellar posterior fossa cyst that is difficult to distinguish from MCM on a CT scan. However, there was no hydrocephalus, compression, or displacement of the cerebellum, vermis, or tentorium cerebelli, making this entity a less likely possibility [12].

This is the first instance of comorbid MDD and OCD in a patient with MCM that has been documented to our knowledge. The presentation coincided with the description of personality changes in CCAS by Schmahmann and Sherman (1998) [2]. While the comorbidities may arguably be incidental, cerebellar involvement in the pathophysiology of these disorders may suggest a direct relationship. Pujol et al. (2004) proposed a possible involvement of the cerebellum in the pathogenesis of OCD, whereby the gray matter volume in the ventral striatum and the rostral cerebellum were significantly increased in patients with OCD [13]. Their findings were further supported by several case studies reporting OCD symptoms in psychotic patients with cerebellar lesions [10,14,15].

A review of structural neuroimaging studies among MDD patients found reduced gray matter density among MDD patients. Structural abnormalities in the pre-fronto-cerebellar circuit were postulated to be a contributing factor to the emotional and cognitive function deficits seen in MDD because of the simultaneous gray matter loss in the left cerebellum and the right dorsolateral prefrontal cortex [16]. Although we are yet to find any cases of MCM in relation to MDD, one case study reported findings on the association between MDD and Dandy-Walker malformation. Kim and colleagues (2013) reported a 33-year-old man with recurrent MDD and impulsive behavior. Brain magnetic resonance imaging (MRI) revealed an enlarged cisterna magna, cerebellar vermal hypoplasia, and dilated ventricles, indicating a Dandy-Walker variant. Their case suggested that the cerebellum could be associated with MDD and impulsive behaviors featuring symptoms such as superficial relationships, impulsivity, recurrent depressive episodes, and resistance to treatment [17].

While neuroimaging may be a valuable modality for assessing, diagnosing, and prognosing psychiatric conditions, even when no neurological presentations are noted, debates about its cost-effectiveness remain equivocal [18]. However, the mounting evidence on the association between psychiatric disorders and underlying brain malformation and developmental problems, as reported by various case reports, calls for the need for thorough neuroradiological evaluation for treatment-resistant psychiatric patients [10,15,18]. In our patient, a significant improvement was observed with the administration of benzodiazepine, which coincided with the case of comorbid OCD-Schizophrenia, as reported by Kani et al. [10].

OCD is characterized by an inability to suppress unwanted intrusive thoughts. These inhibitory deficits are mediated by abnormalities in cortical GABA (gamma-aminobutyric acid) [3]. GABA is the primary inhibitory neurotransmitter in the central nervous system, and GABAergic neurons are primarily responsible for its synthesis [19]. GABA neurotransmission disorder has been observed in OCD via cortical inhibitory processes [19]. It is well known that benzodiazepines increase GABAergic function and inhibitory processes by GABAergic neurons, of which several types work in both the cortex and the cerebellum nuclei [20]. In our case, the presence of MCM, MDD, and OCD symptoms may coincide or be influenced by MCM, potentially leading to obsessions and cognitive symptoms [4].

## Conclusions

Organic neurological lesions, such as cerebellar abnormalities, may contribute to treatment-resistant psychiatric disorders, as evident in this case. We recommend utilizing neuroimaging modalities to investigate organicity. Furthermore, considering benzodiazepines as a potential treatment option for managing the patient’s obsessive-compulsive features is worth exploring due to their potential effectiveness.
